# District decision-making for health in low-income settings: a qualitative study in Uttar Pradesh, India, on engaging the private health sector in sharing health-related data

**DOI:** 10.1093/heapol/czv117

**Published:** 2016-09-01

**Authors:** Meenakshi Gautham, Neil Spicer, Manish Subharwal, Sanjay Gupta, Aradhana Srivastava, Sanghita Bhattacharyya, Bilal Iqbal Avan, Joanna Schellenberg

**Affiliations:** ^1^IDEAS Project, London School of Hygiene and Tropical Medicine, London, UK,; ^2^Impact Partners in Social Development, Malviya Nagar, New Delhi, India,; ^3^Public Health Foundation of India, Vasant Kunj Institutional Area, New Delhi, India

**Keywords:** Data sharing, health management information system, public–private engagement, public health sector, private health sector, MNCH data

## Abstract

Health information systems are an important planning and monitoring tool for public health services, but may lack information from the private health sector. In this fourth article in a series on district decision-making for health, we assessed the extent of maternal, newborn and child health (MNCH)-related data sharing between the private and public sectors in two districts of Uttar Pradesh, India; analysed barriers to data sharing; and identified key inputs required for data sharing. Between March 2013 and August 2014, we conducted 74 key informant interviews at national, state and district levels. Respondents were stakeholders from national, state and district health departments, professional associations, non-governmental programmes and private commercial health facilities with 3–200 beds. Qualitative data were analysed using a framework based on *a priori* and emerging themes. Private facilities registered for ultrasounds and abortions submitted standardized records on these services, which is compulsory under Indian laws. Data sharing for other services was weak, but most facilities maintained basic records related to institutional deliveries and newborns. Public health facilities in blocks collected these data from a few private facilities using different methods. The major barriers to data sharing included the public sector’s non-standardized data collection and utilization systems for MNCH and lack of communication and follow up with private facilities. Private facilities feared information disclosure and the additional burden of reporting, but were willing to share data if asked officially, provided the process was simple and they were assured of confidentiality. Unregistered facilities, managed by providers without a biomedical qualification, also conducted institutional deliveries, but were outside any reporting loops. Our findings suggest that even without legislation, the public sector could set up an effective MNCH data sharing strategy with private registered facilities by developing a standardized and simple system with consistent communication and follow up.

Key Messages

- Public health management information systems are an important tool for planning and monitoring public health services, but may lack information from the private health sector.
- Data sharing for maternal and newborn care services was weak in Uttar Pradesh, although most private facilities did maintain basic records related to institutional deliveries and newborns.
- Barriers to data sharing included gaps in the public sector’s data collection systems, data utilization and communication; and private providers’ fears of disclosure and perceptions of the level of work involved.
- The private sector’s willingness to share public health data can be harnessed by the public sector through increased communication, trust and relationship building, and establishing a sustainable system for data collection and synthesis.

## Background

Health information is an essential constituent of a health system. Policy makers and health administrators require health information for planning and monitoring health services and tracking health indicators. They require information on infrastructure and human resources, service delivery, health financing and management and the disease burden (Stansfield 2005; Raban *et al.* 2009). In many low- and middle-income countries, national health surveys like the Demographic and Health Survey or the India District Level Health Survey, provide some of this information with varying periodicity and the more regular and routine health data are available through health management information systems (HMIS) in the public sector (AbouZahr and Boerma 2005; Pandey *et al.* 2010).

There may be substantial gaps in the public sector HMIS including incomplete and poor quality data, data duplication and overload and gaps in data management and utilization (Simba 2004; Raban *et al.* 2009; Bhattacharya *et al.* 2012). Another major gap is limited information sharing between the private health sector and the public sector. This gap is of special concern for India, where the private for-profit sector represents more than two-thirds of human resources for health and provides a substantial proportion of health services, including maternal and child health services (Government of India 2005). These data relate to the formal private biomedical or allopathic sector and it is important to make this explicit because India also has professionalized traditional medical systems such as Ayurveda and Unani, [Department of Ayurveda, Yoga and Naturopathy, Uanani Siddha and Homeopathy (AYUSH)] as well as a vast informal private sector apart from the formal allopathic private sector (Government of India 2005). The private formal allopathic sector is the more dominant and the focus of this article, but there is almost no information available in the public sector HMIS on its infrastructure, human resources or service delivery (Raban *et al.* 2009).

Availability of private sector data in the public health information system can contribute to improving health outcomes by providing more comprehensive mapping of the health sector, including the size, composition, behaviour and practices of the private sector thereby enabling better health systems planning (Sood *et al.* 2011). Such comprehensive data can also inform policy advocacy, (Manandhar *et al.* 2008) social mobilization (Suresh 2011) (e.g. for immunization) and strengthen communication and referrals for improved health services. For example, the Integrated Disease Surveillance Project in India encourages private practitioners to report any suspected disease outbreak among humans as well as in animals (Suresh 2011). Private sector involvement in the Revised National Tuberculosis Control Programme in India has, through improved drug supplies, and improved reporting and referral systems, led to higher case detection and treatment rates (Floyd *et al.* 2006). Information sharing also represents a means for the private sector to be more engaged in public health goals and outcomes and, in becoming part of a larger inter-sectoral collaboration at local level, ultimately resulting in improved public relations between the different sectors involved in health (Manandhar *et al.* 2008).

The absence of a regulatory framework may be one of the major reasons for the private sector’s lack of interest in sharing health information. This is especially true in India where the growing private sector remains weakly regulated. Yet health initiatives such as the World Health Organization’s Public-Private Mix DOTS (Floyd *et al.* 2006) and the Integrated Disease Surveillance Programme in India. Suresh (2011) show that a public-private health information partnership can be created, even without a regulatory framework. These examples are indicative of a latent willingness in the private sector to share health data which could be harnessed through a better understanding of workable strategies. Uttar Pradesh (UP) is the most populous state in India, with one of the highest maternal mortality ratios of 258 per 100 000 live births and the highest infant mortality rate of 68 per 1,000 live births in the country (2012–2013 data) (Government of India 2014). The private sector in UP provides 90% of treatment for acute illnesses, 80% for chronic conditions and accounts for around 18% of institutional deliveries in the state. Institutional deliveries constitute 56.7% of all deliveries in the state, 39% of these are in government facilities and 17.6% in private ones (2012–2013 data) (Government of India 2013). However, there is poor health record keeping and information sharing by the private sector. A health facility survey carried out during 2013 in 25 districts of UP reported that half of the 731 mapped private facilities providing institutional deliveries did not maintain any relevant records (Karnataka Health Promotion Trust and University of Manitoba 2013). Thus, there is a need to build a greater understanding of how the private for-profit sector in UP can engage in an information sharing collaboration with the public sector.

This article is part of a series of four on the district data for decision-making for health in low-income settings. The first reports the feasibility of establishing a data-informed platform for health to support district data for decision-making in India, Nigeria and Ethiopia; the second reports a systematic literature review of the use of district data for decision-making in low-income settings (Avan *et al.* 2016, Wickremasinghe *et al.* 2016). The third article in the series (Bhattacharyya *et al.* 2016) shows the huge untapped potential of public and private sector data for decision-making in India and Ethiopia; and in this final article, we describe a study to assess the extent of data sharing by the *formal private allopathic for-profit health sector* at state and district level in UP and to a certain extent at national level in India; analyse the barriers to data sharing; and identify the key efforts required for engagement with the private sector.

## Methods

This was a qualitative study conducted during 2013–2014, focusing on the formal private health sector. In March–April 2013, we conducted 20 key informant interviews primarily at national and state levels, in Delhi (10 interviews) and Lucknow (seven interviews), respectively and three interviews at district level in Rae Bareli. We also held a national level group discussion in Delhi in April 2013, with 10 participants. Interviewees included senior representatives in government health information repositories, accreditation bodies, academic institutions and professional associations of medical practitioners (Table 1). In 2014 (April–August), we conducted another round of 54 interviews primarily at district level; these included private commercial health facilities in two districts with a bed strength ranging from 3 to 200 beds (25 interviews—see Table 2), key stakeholders from the state and district health departments (18 interviews) and from district level medical associations and non-governmental organizations (11 interviews). Respondents were selected based on their leadership and knowledge, involvement in data processes and engagement with maternal, newborn and child health (MNCH) services. For this second round we selected two districts, Hardoi and Allahabad, from the 25 districts that were being supported at the time of this study by the UP-Technical Support Unit (TSU), a donor funded programme of assistance to the UP state government. Allahabad had the largest number of private *tertiary* facilities and Hardoi had the largest number of private *primary* facilities, according to a facility survey conducted by the TSU in 2013.

**Table 1:** . Key informants and their representative organizations (2013 interviewees)

Level	Stakeholder category	Organizations included	No. of key informants
National	Key policy making bodies	Ministry of Health and Family Welfare; Planning Commission	2
	Public-private partnership in human resource training	Public Health Foundation of India	1
	Accreditation body	National Accreditation Board for Hospitals and Healthcare Providers (NABH)	1
	Health information repositories	Central Bureau of Health Intelligence (CBHI); National Health Portal	2
	Professional associations	Federation of Obstetrics and Gynaecological Societies of India (FOGSI); Indian Academy of Pediatrics (IAP)	3
	Technical support institution	National Health Systems Resource Centre	1
State	Professional associations	FOGSI; IAP; UP Nursing Homes Association; Lucknow Obstetrics and Gynaecology Society; Practicing Gynaecologists’ Association	5
	Health information repository	National Health Mission, UP	1
	Technical support institution	State Institute of Health and Family Welfare	1
District	Professional associations	UP Nursing Homes Association; IAP	3
		Total	20

**Table 2:** . Private facilities selected for interviews in Hardoi and Allahabad districts (2014 interviewees)

Volume of deliveries/month	Hardoi (14 facilities)		Allahabad (11 facilities)	
	Reporting	Non reporting	Reporting	Non reporting
High	Facilities: 2	Facilities: 2	Facilities: 2	Facilities: 3
	Beds: 18, 100	Beds 20, 100	Beds: 200, 200	Beds: 3, 20, 30
	Deliveries:100, 144	Deliveries 95, 100	Deliveries:100, 200	Deliveries:100, 40,100
Medium	Facilities: 4	Facilities: 3	None	Facilities: 4
	Beds: 20, 20, 20, 20	Beds: 5, 10, 60		Beds: 15, 15, 15, 10
	Deliveries: 15, 10, 10, 20	Deliveries: 15, 8, 25		Deliveries: 12, 10, 12, 10
Low	None	Facilities: 3	None	Facilities: 2
		Beds: 10, 20, 30		Beds: 10, 10
		Deliveries 1, 2, 2-8		Deliveries 1, 10

### Selection of private health facilities for interviewing (see Table 2)

From among those facilities that provided MNCH services, especially institutional deliveries, we selected facilities with delivery loads varying from 1 or 2 to 100 deliveries per month and among these we selected a few that were providing some records of institutional deliveries to the district health department and those that were not (see Table 2).

We first reviewed the TSU’s facility data for both districts and identified those that performed institutional deliveries. Next, through discussions with data staff in the Chief Medical Officer’s office (in the district health department), we identified those facilities that were already providing some rudimentary records of institutional deliveries (e.g. numbers of deliveries per month). We then shortlisted the blocks where most of our facilities of interest were located, visited the government health facilities in these blocks to confirm our selection and validated the information obtained by talking to some of the local pharmacists, pathology centre staff and staff at other local clinics in the area. We then visited the selected facilities and interviewed selected staff members, after seeking consent and scheduling appointments.

In both districts, key informants in the public sector informed us that a large number of institutional deliveries were being conducted in unregistered facilities too. These key informants provided us the names and coordinates of two such facilities that were popular and had high estimated delivery loads. We visited these areas and confirmed the presence and popularity of the two facilities by talking to local pharmacists and a few community members. With their help we obtained the exact locations of these facilities, visited them and interviewed the staff.

### Interview topics

Interviews were based on topic guides developed for different categories of stakeholders. Major areas of enquiry were: (a) roles and functions of organizations with respect to the private health sector; (b) informants’ views and knowledge about current data sharing by the private health sector, and barriers and enablers to these; and (c) recommendations for a sustainable public-private data sharing strategy. The group discussion in Delhi focussed on (c) above. With the private health facilities we queried the existing status of all the MNCH services-related records that they maintained and shared with the district health department.

Data were captured using detailed field notes and analysed qualitatively using a framework approach involving drawing out both *a priori* and emerging themes. Field notes were organized in a matrix under the main themes and sub-themes and analysed for common as well as divergent views, areas of conflict and disagreements, and for detailed accounts of recordkeeping and data sharing.

### Ethical approval

We obtained ethical approvals from the Health Ministry Screening Committee of the Indian Council of Medical Research and the corresponding author’s institute. Informed verbal or written consent was obtained before commencing each interview and the individual interviews took place in private spaces to maintain confidentiality.

## Findings

### Data sharing: current national context

#### Public sector experiences with obtaining private sector health data

There was very limited MNCH data sharing between the private and public health sectors at any level: national, state or district. National planning and policymaking bodies, such as the Planning Commission and national health data repositories, such as the Central Bureau of Health Intelligence (CBHI) and the National Health Portal, had only partially succeeded in receiving data from the private sector. The CBHI faced difficulties in obtaining private sector health data on a regular basis; thus most of their annual *National Health Profile* was based on public sector data. CBHI’s private sector health information was limited to examples of public-private partnerships on the agency’s Health Sector Policy Reforms Options Database (www.hsprodindia.nic.in). The National Health Portal, another public sector initiative, was an effort to address the private sector’s lack of responsiveness to data sharing by creating an ‘*attractive and easy space’* for the private sector to utilize and contribute to (www.nhp.gov.in). It was designed as a one-stop online portal for all information related to health, for health care users and providers, and was launched a few months prior to this study in November 2013. Some private facilities including owner operated clinics as well as single and multispecialty hospitals had shared their contact information on the portal at the time of this study. The National Accreditation Board of Hospitals and Healthcare Providers (NABH) was an autonomous national agency providing accreditation to private facilities as a self-regulatory initiative. NABH accreditation was useful to many private facilities and they were willing to comply with NABH’s reporting requirements on a ‘*variety of areas including clinical, service related and infrastructural components’*. Some of these were mandatory and others optional. NABH analysed these data and ‘*provided feedback to providers’*; however, these data were not available in the public domain.

#### Private sector initiatives to promote data sharing

Professional medical associations including the Federation of Obstetric and Gynaecological Societies of India (FOGSI), the UP Chapter of Obstetricians and Gynaecologists, district level FOGSI affiliates such as the Lucknow Obstetrics and Gynaecology Society and the Indian Academy of Pediatrics (IAP), played an important role in bridging the gap between individual private sector providers and the government through public health activities:

*We worked with the Government polio immunisation campaign (Pulse Polio) and encouraged our members to also follow the same schedules. We participated in the immunisation schedule development* (representative of a professional specialists association).

These bodies displayed a growing understanding of the need for the private sector to maintain data on public health activities, especially in their newer, public health oriented initiatives such as adolescent clinics and Diabetes in Pregnancy clinics, and in registers of critical diseases of public health significance, like childhood pneumonia and diarrhoea. They used these data to provide timely feedback to the reporting practitioners, which also served as a motivational strategy:

*The person contributing the data feels acknowledged… understands the results from their reporting and also the importance of the data contributed…this further motivates them* (leader of a professional specialists’ association).

FOGSI promoted self-regulation among its membership by increasing members’ awareness of standard guidelines and the need to comply with these guidelines, including reporting on public health problems such as cervical cancer, eclampsia and maternal mortality. Compliance however was poor and one FOGSI respondent articulated the government’s potentially important role in improving this situation:

*Only 10% of members are actually reporting… Maharashtra government has taken it up to pressurise compliance on the registries, as the potential data would be quite useful* (leader of a specialists’ association).

For some public health initiatives implemented by associations, intermediary bodies were entrusted with the task of data collection:

*For our recent project ‘Helping Mothers Survive’, JHPIEGO is carrying out the [monitoring and evaluation], so they would be collecting and maintaining data* (representative of a specialists’ association).

### Data sharing: current state and district level context

The health directorate situated in the state government, as well as the state and district units of the centrally funded National Health Mission (NHM) (Ministry of Health and Family Welfare 2013) were engaged in collecting and maintaining public health data through two parallel systems. The health directorate continued with a paper-based health reporting system: manually collected data flowed from block level facilities to the district Chief Medical Officer’s office where it was consolidated into a district Monthly Progress Report (MPR) every month. Under the NHM, central government had introduced a computerized data collection and reporting system across all states—HMIS. HMIS data were entered online by block level facilities, eliminating the need for manual consolidation.

Most of the private facilities we visited, especially those that were registered and licensed for conducting ultrasounds and abortions, were used to some form of rigorous record keeping and data sharing with the Chief Medical Officer’s office in the district health department. In both districts it was possible to establish the number of facilities that were registered for ultrasounds and for medical termination of pregnancy (MTP). These services were closely supervised and monitored under Indian law, via the Pre-Conception and Pre-Natal Diagnostic Techniques Act (PCPNDT Act) and the Medical Termination of Pregnancy Act (see Box 2). All facilities registered for these services in Hardoi and Allahabad submitted meticulous and standardized records on these services (see Table 3) on a fixed date every month. However, no such information was available for facilities that performed deliveries, as this service did not require a separate registration process.

**Box 1**. The private commercial health sector in UP

The private sector in UP is autonomous and self-financed, as in the rest of India. It consists largely of solo doctor clinics providing primarily outpatient care, and single-speciality and multi-speciality hospitals providing both outpatient and inpatient care.

According to data obtained from the State Medical Council of Uttar Pradesh in 2012, there were 15 private medical colleges in the state compared with 12 government ones and the number of hospital beds in the private sector (208 000) far exceeded the number of beds in the public sector (63 950).

A study conducted by IDEAS in 2012 in two districts of UP recorded 45 public sector facilities (primary, secondary and tertiary) and 196 solo proprietorship allopathic clinics, 1103 non-allopathic (ayurvedic/unani/homeopathic) clinics and 71 hospitals in the formal private sector. These were registered with the district health department.

Source: IDEAS 2012.

Box 2. Potential incentives for private sector stakeholders to share data

The Pre-Conception and Pre-Natal Diagnostic Techniques (Prohibition of Sex Selection) Act, 1994 (PCPNDT Act) and the Medical Termination of Pregnancy (Amendment) Act, 2002 (MTP Act).

The PCPNDT and the MTP Acts are implemented quite strictly in India with the objective of arresting the declining sex ratio of girls:boys by banning identification of the sex unborn children through ultrasound, and sex selective abortions. An action plan for the PCPNDT was put in place under the National Rural Health Mission's Save the Girl Child programme. Both Acts require compulsory registration (with renewals) of facilities that provide either ultrasonography or abortion services. These facilities have to be open to periodic inspection visits by the health department, and to maintain and submit essential records related to the relevant services. Failure to do so can result in penal action including fines as well as imprisonment.

Source: The Pre-Conception and Pre-Natal Diagnostics Techniques Act, 1994.

The Medical Termination of Pregnancy Act, 1971.

**Table 3:** Facilities in Hardoi and Allahabad reporting on ultrasounds, MTPs and deliveries to the district health departments

District	Number of private facilities registered	Reporting on ultrasounds (PCPNDT Act)/total registered under the Act	Reporting on MTPs/total registered under the Act	Reporting on deliveries
Hardoi	34	19/19	8/8	7
Allahabad	283	205/205	11/23	N/A

*Source:* Chief Medical Officers’ records in Hardoi and Allahabad districts.

Private facility owners also went through a gruelling procedure to set up and register a new facility:

*In order to start a private health facility, 26 different licenses and No Objection Certificates are needed from various departments like Development Authority (for land), Municipal Corporation, CMO’s Office, PCPNDT Act, Radiation, MTP, Labour Department, Provident Fund, electricity, taxation, Pollution Control Board and fire being some of the main ones. Most of these are renewed annually and some after three years* (owner of a private facility).

Registered private facilities maintained records of deliveries and newborns in different formats like out-patient registers, in-patient registers, operation theatre registers, or labour room records. This information varied from hospital to hospital and could include: (a) mother’s name, age, address, dates of admission and discharge, normal or caesarean delivery, order of birth; (b) newborn’s gender, birth weight, born alive or dead, born full term or pre-term and time of birth. However, most private hospitals did not share these data with the district public health department.

A few private hospitals shared some data on deliveries and newborns with the public sector, but in varied and non-standardized formats. They had been doing this for many years after receiving a letter from the health department. We estimated the total number of facilities that reported these data in Hardoi district (see Table 3) by reviewing all the facility records in the CMO’s office. This was not possible in Allahabad where there was a much larger number of secondary and tertiary facilities than in Hardoi.

Public sector facilities at the block level (primary and community health centres) had developed their own different methods for collecting this information ranging from paper forms, to obtaining data by telephone. These data however were integrated only into the district health department’s Monthly Progress Report, and did not appear in the HMIS of our study districts (for the month previous to this study).

We learned more about the gaps in private sector HMIS reporting from data management staff:

*As of now, 39 out of 75 districts submit this report on the web HMIS. The remaining districts have requested for disabling this feature as they have admitted they cannot receive and process data from the private sector. The 39 reporting districts submit a monthly report but these reports (of private facilities) are largely incomplete and report on very few indicators* (public sector data manager).

We also found that a large number of institutional deliveries within the private sector could be taking place in unregistered private facilities, managed by providers without an appropriate medical qualification or formal training in maternity care. An essential criterion for facility registration was that there should be at least one doctor with a graduate degree in biomedicine (Bachelor of Medicine and Surgery or the MBBS degree as it is called in India). Facilities without this criterion could not register. We could not obtain reliable estimates of the total numbers of unregistered facilities, or the proportion of deliveries being conducted in these, but the two that we visited reported high case loads of 100 deliveries per month. Being unregistered, these facilities were entirely outside any reporting frameworks.

*Most nursing homes operating in this area do not have qualified doctors. Many of these are absolutely unqualified. So they do not want to come on record* (member of staff at a block level government facility).

## Barriers to improving the situation of MNCH data sharing

### Legal barriers

#### Lack of a binding legal framework

Several public sector stakeholders, especially at national and state levels, were of the view that the private sector would not share any data voluntarily or without coercion and that legislation was necessary to make data sharing mandatory: ‘*First the private sector should come under a common regulatory framework through the Clinical Establishments Act; without that any engagement strategy would not be effective.*’ However, there were alternative viewpoints too, that acknowledged and tried to harness the varying needs and interests of the private sector through creative techniques rather than enforcement. Efforts made by the National Health Portal and the NABH were two examples of innovative strategies based on alternative thinking.

Private sector stakeholders perceived the legal barrier differently. While legislation did not figure prominently in their narratives, they did articulate the need for a certain amount of enforcement from the government to ensure private providers’ compliance with data sharing. Some private sector respondents expressed this as a communication gap (described in the next barrier) rather than an enforcement issue.

*We have no idea if there are any laws which mandate private sector to submit data. However, [the Chief Medical Officer] has all powers. If he wants to get data, we will have to provide data* (owner of a private health facility in Hardoi).

#### Existence of unregistered facilities

We could not get official estimates about the numbers of these facilities, but from the responses of a few public and private sector key informants, we understood that these facilities were not registered because they did not meet the essential criteria of having a doctor formally qualified in modern medicine or biomedicine on the rolls. Of the two facilities we visited, one was managed by informally trained nurses and the other by a practitioner trained in an indigenous medical system. Both facilities had limited contact with the formal health system and did not maintain any records;

*We avoid keeping records because the government can catch us if they find records with us. If there are no records, there is no evidence of what we have done in the past* (owner of an unregistered facility in Hardoi).

However, both facilities were willing to engage with the health system and to submit any required data in the hope that the public sector would recognize them, give them registration and help them to enhance their services.

### Lack of official communication or engagement

Failure to receive official communication from the public sector emerged as an important reason why many private facilities were not sharing any reports or data on deliveries and newborn care. A few respondents from the older and more established private facilities recalled having received one communication about seven or 8 years previously, and as a result, a few facilities had started reporting. But the public sector neither repeated this communication with newer facilities, nor followed up in a sustained way with those facilities that did not report. This communication failure had proved to be a significant barrier in data sharing:

*We do not share it [data] because no one has ever asked for any data from us. The (community health centre) is just opposite this nursing home but they have never visited us. They always call us for help whenever there is a critical case and they want us to take the case from them and either treat at our nursing home or refer and transport the case in our ambulance* (a private facility staff member in Allahabad).

Another related factor was the limited scope for formal engagement of the private sector in public health planning and goal setting at district level. There were few platforms for bringing together the two sectors regularly. Those that did exist, like District Health Society meetings, had very limited participation from the private sector.

### Mutual mistrust and attitudinal problems

Mutual mistrust emerged in the narratives of both public and private sector respondents at all levels. Public sector respondents’ common view was that the private sector was unwilling to share any data, while private sector respondents complained of government mistrust and lack of engagement:

*Government treats us like local grocery traders and not as professionals. They think that we are minting money. No matter how much we speak the truth, they always doubt us and our intentions* (a private facility owner in Allahabad).

*The Government needs to do a lot more to constructively engage with the private sector. For that it is imperative that first a climate of trust is built up; right now that is missing* (representative of a professional medical association).

Some private sector respondents expressed dissatisfaction with the data receiving staff in the health department offices:

*We are friendly but they are not. They are not friendly to the staff that go to submit the reports… they have an attitude problem* (owner of a private facility in Allahabad).

However, several private sector stakeholders also accepted that it was difficult to obtain information from the private sector and FOGSI and IAP faced difficulties in getting their members to comply with data reporting. One reason could lie in the type of services and orientation of the private sector:

*Until about five years ago, there was no focus on public health by FOGSI, except for isolated, one-off programmes. But there was a change when FOGSI entered into partnership with JHPIEGO for [emergency obstetric care] guidelines. JHPIEGO facilitated the development of the public health mindset within FOGSI* (leader of a specialists’ association).

Private sector respondents also suggested that it might be difficult to motivate the more senior and experienced practitioners within well-established practices, and they could negatively influence their junior colleagues:

*The younger cadre, even if wanting to comply, is pressured to follow the more established practitioners, who are often resistant to change* (representative of a specialists’ association).

### Lack of standardized formats and data collection systems

A few private facilities submitted monthly reports on institutional deliveries conducted in their facilities, but aside from some common features like the mother’s details, type of delivery, date and time of delivery, the reports varied from facility to facility. Most reports were manually compiled, but we also found a few instances of very well presented computerized reports. The public sector had not provided any standardized forms for receiving the required data, and block level public facilities used different methods to collect this data (for example, verbal estimates on phone or in person) from the few private facilities that reported it regularly.

This process was part of the state government’s paper-based MPR system but did not show up in the computerized HMIS promoted through the NHM. Both systems existed in parallel in the public sector and were operational at the time of this study, but the MPR was expected to be phased out gradually. Both systems had different data entry staff, different formats and different requirements for private sector reporting at source. The current HMIS did not include any data from the private sector in either district, although we learned that 39 out of 75 districts were reporting some data on the web-based HMIS, even though it was incomplete and irregular. In general, the MPR system was better established than the newer HMIS and the district HMIS data entry staff were not well informed about private sector data reporting in the HMIS.

#### Inadequate coordination and management

Private as well as public sector stakeholders were of the view that the lack of a central private sector coordination body in the district health department was a significant barrier in dealing comprehensively with private sector issues including timely data sharing. Additionally, there had been weak public sector management of the HMIS from state to district level and this could continue to be a barrier.

*The HMIS unit at state level trained the district level functionaries and expected that they would train the block level functionaries in data management. However, this did not happen and also due to third party engagement in recruitment, there was a high turnover of staff which led to a lot of trained people leaving the job* (a state level data manager).

#### Resources and effort required for data sharing

Several private sector respondents explained that data capturing and sharing was a time consuming and technical task that required certain systems to be in place, including hardware and software, human resources and other logistics. Not all private sector providers had enough resources to manage this, and they already felt burdened by other paperwork required for the government system such as renewal of licences:

*They have reduced us to clerks. There’s too much paperwork. The biggest barrier is that we’ll have to sit and compile. I have just five beds and one admission at a tim* (a private facility owner in Allahabad).

Some respondents pointed out that initiatives like the polio eradication campaign had successfully developed good data sharing mechanisms because of good coordination and engagement systems, developed by the public sector that included simplified formats and data collection processes, and appropriate incentives including travel allowances and supplies.

#### Mismatched interests and lack of motivation

Private hospitals maintained records based on their own unique needs and requirements, and these were usually focused on curative services rather than preventive ones. It would require some effort for private facilities to align this record keeping with the requirements of the public sector HMIS. A few stakeholders observed that the absence of any incentives for record keeping, or provision of commodities by the public sector (e.g. vaccines) was another barrier to efficient record keeping and sharing. Furthermore, hospitals that performed a very small number of deliveries thought that reporting these would be a big effort, as well as unnecessary.

Government doctors in private services could also pose a key barrier to accurate reporting:

*Some government doctors also practiced privately and some had their own private facilities. As reporting on their services would expose a conflict of interest, these facilities and providers would not be motivated to report appropriately* (a private sector stakeholder).

In the public sector, limited feedback on private sector reporting by state health department officials to district officials was a motivational barrier.

### Perceived limited capacity for data use by the public sector

Private, as well as public sector stakeholders were of the view that there was limited ability in the public sector to analyse any new data coming in, and limited computers and computing skills. A few public sector stakeholders expressed concern that if data started coming in, the government might not be equipped to handle it.

As of now streamlining the government reporting system is a big challenge with the government, particularly the timing and quality of reports. Private sector reporting, therefore, is not a current priority with the government.

*Government sector may not have the willingness or capacity to receive large amounts of data from the private sector and process it for integrating into the government system* (data manager at state level).

Private providers frequently complained that once submitted, their reports were discarded without being utilised for any policy or planning.

…*There is no processing, or analysis, or strategy setting. They just throw away the data… they don’t use it in any way* (a private facility owner in Hardoi).

Some private sector stakeholders stated that the public sector was discomfited by any data that could show their district in a bad light and might therefore not accept some data that could draw public attention to an adverse situation (for example excessive newborn deaths).

*Government sometimes does not want to accept private sector data, particularly on vector borne and water borne disease [e.g. dengue fever and diarrhoea]. This is because it reflects government’s failure to control these diseases* (head of a state level non-governmental organization programme).

### Private stakeholders’ fears of information disclosure and harassment

A few private sector stakeholders expressed fears that the health department might disclose service-related data to the income tax department, who would then harass private facilities about their tax returns. Many respondents were also worried about government harassment related to the reporting of mortality or complicated cases:

…*If we report a stillbirth then they ask us why this happened here. They do not understand that patients come here in distress… like when the dai [birth attendant] has given up and there is either breach, or placenta previa, or obstructed labour*… (private facility respondent).

*If we send some data, they may send a notice that what happened to this patient… then we have to go and collect that information and show the full record… then we have to go and search for that patient… this creates extra work for us* (private facility respondent).

### Enablers for improving the situation of MNCH data sharing

#### Private stakeholders’ general willingness to maintain and share records

Most private facilities we visited, even those that maintained only basic records, were not averse to maintaining and sharing the required MNCH data. They were willing to submit these data if the health department asked them to do so.

*We believe in submitting what is being asked for. We submit the required data (ultrasounds) because we are being asked. I feel that it is our contribution to provide what information is being asked for* (private facility respondent in Hardoi).

Associations of general medical practitioners, paediatricians, and gynaecologists at national and state levels were also willing to cooperate by communicating the data sharing requirements to their members:

…[*the association] has an ethical and legal committee that can discuss the modalities of data submission to government system and convince their members* (office bearer of a state level association).

#### Perceived importance of communication from the public sector

Private facilities attached a lot of importance to official communication from the health department or the office of the Chief Medical Officer. Private hospitals that submitted any type of records (including for ultrasounds and abortions) recalled receiving an official communication as the very first step in kick starting the submission process. In addition, those hospitals that were not submitting any records at the time of this study said that they would have done so if they had received an official communication. So, in the perception of private stakeholders, a communication from the health department carried weight.

#### Basic systems in place for data maintenance

Bigger and more established private hospitals especially, needed to maintain meticulous records of their services as a safety precaution against medico-legal cases. All hospitals that performed deliveries needed to give some proof of birth to their clients and so maintained these records for at least a year, as sometimes clients could come back later asking for the information. Most private facilities were able to make available some staff for keeping records. These were usually multi-tasking staff (e.g. nurses, ward boys) who did other work in the facility as well as looking after records. This self-need for record keeping and the systems presently available could serve as a foundation to further improve record keeping and data sharing.

#### Birth data cannot be hidden

Although fearful of income tax disclosures, private stakeholders said that since they had to provide birth proof to all their patients, it would necessitate accurate birth reporting to the health department as well. Another related enabling factor was the existence of Birth and Death Registration legislation that provided legal cover for mandatory reporting of all births and deaths that occurred in private facilities. As all births have to be registered with the appropriate village or urban bodies under this Act, the health department could triangulate data from these sources to validate data on institutional deliveries from private facilities.

## Stakeholders’ recommendations for developing an engagement strategy

### 1. Increased communication and engagement between the public and private health sectors

#### Fostering rapport and sensitization of private and public stakeholders

Private sector respondents recommended that strategically it was better for the public sector health department to work through groups of private providers (such as professional associations of medical practitioners), rather than directly with individual providers, so a first step could be to identify appropriate forums and support them in creating and maintaining good interpersonal relationships through regular interactions.

At the state and district levels, there was limited awareness about the importance of data sharing among different stakeholders. Therefore a key task would also be to create awareness of the concept and systems for data sharing and their significance for decentralized public health decision-making. This could be done through meetings or sensitization workshops by the public sector.

#### Identifying champions to catalyse data sharing

Engaging the most responsive private sector players initially would inspire others to follow their example. A few respondents were of the view that all private providers would not cooperate equally and some might drop out due to lack of interest or time. Therefore it was important to identify the most socially oriented, or more enthusiastic doctors, in the district and start with them. Similarly, champions for the engagement strategy would also need to be identified in the public sector at state and district levels.

#### Role of official communication and sustained coordination by the public sector

The public health department would need to take the lead to ensure proper communication and follow up with all private health facilities. Private sector respondents recommended that the district health department set up a coordinating body to oversee all issues related to the private sector. They felt that the government should take responsibility for ensuring data sharing; it should not be left to the choice of the private sector to share data or not. The success of the polio campaign was described as an example of good coordination by the public sector:

*For eradication of polio, a strong network of reporting units (private hospitals) and informers was established. Each private hospital was closely monitored to ensure reporting (even if there were no cases i.e. ‘0’ reporting). Any suspected case was then tracked back along the referral chain and followed up* (a senior public sector state health official).

The implementation of data sharing systems for ultrasounds and abortions also provided important lessons in good communication and follow up by the public sector.

### 2. Design a user friendly system

#### Introduce simplified data formats, and collection and analysis processes

The public sector should develop user friendly formats in consultation with private bodies; prioritize the most critical data; and create a simple system with online data entry provision. As private providers did not have much time for laborious data recording and may also not be willing to share all their data, it would be useful to seek their inputs into data formats and the acceptability of the data. One respondent explained that the data to be collected should only be of public health importance and should not have medico-legal implications (e.g. injury or accident cases). The responses could be coded and include ranges instead of exact numbers. Reliable modalities for regular data collection, online or through appointed data collection staff, should be established. A few respondents also suggested that the system should be useful for the private providers as well:

*Create systems that enable the private sector also to use their own data for different purposes including planning, assessing their own performance and publications* (a senior representative of a specialists’ association).

### 3. Capacity building of the private and public sectors

Both public sector and private sector key officials would require technical assistance in data collection and management for setting up the system. This could be through orientation, training and periodic follow-up support. One respondent recalled the process employed for the PCPNDT reporting:

*In the beginning we could not complete some columns…so they had meetings in Hardoi to explain …some organisation [non-governmental organisation] in association with the health department came to explain* (private facility respondent, Hardoi).

### 4. Address the private sector’s fears

The government needed to reassure private facilities that they would not be harassed over any data and the information would not be disclosed to the income tax department. Data confidentiality issues would need to be worked out:

*Government should provide adequate risk cover to the private sector for any issues after sharing the data* (senior officer of a state level association).

*One is maintaining the confidentiality of data; whatever data is being submitted to government should be confined to them only. Government should not share the same with the income tax department, which probably is the main fear. Anyone can calculate the earning/income of the health facility by merely multiplying the numbers by rates for getting the idea of annual or monthly income of a facility* (private facility owner, Allahabad).

### 5. Encouragement and motivation

Respondents suggested a variety of incentives to reward and encourage private providers (See Box 3). The public sector could offer simple incentives—financial and non-financial (such as certificates of recognition) to motivate private health facilities to share MNCH data. Disincentives would also be useful (like a penalty for not complying with submission, as is the case in not reporting ultrasounds). However there were also a few who disagreed with the need for incentives:

Box 3. Potential incentives for private sector stakeholders to share data

- Certificates of participation
- Membership of associations or names in publications
- Tax exemptions
- Performance based incentives such as for every completed immunisation
- Provision of logistics and supplies, such as free or subsidised drugs, equipment, vaccines and equipment
- Some privileges like extended supply of electricity without power cuts
- Information and communication material, continuing medical education
- Transport allowance and other cash incentives to reimburse travel and time
- Sponsored exchange visits

*I personally think that we work for the community and are doing it with passion and dedication. We do not need motivation from the government or any kind of incentives…not at all required* (private facility owner, Hardoi).

## Discussion

We found that private for-profit health facilities were not resistant in principle to data sharing with the district public health department. In fact those facilities that were registered and licensed by the health department for ultrasounds and abortions routinely maintained and shared meticulous records on these services. These services are governed by the PCPNDT Act and the MTP Act in India, legislations that mandate regular reporting by facilities, and provide implementation guidance for the state and district level health authorities as well. This explains to a large extent the effective data sharing for these services. Another instance of good data sharing had occurred during the polio campaign when the public sector had implemented a well-coordinated effort for seeking prompt information on polio cases from private facilities.

As health data sharing is not currently legislated for other services in the private sector such as institutional deliveries and newborn and child health services, it was not as well developed as for ultrasounds and abortions. Still, a rudimentary system was in place for block level public health facilities to collect data on deliveries and newborns from a few private facilities, using different methods and formats. There were two different health data management systems implemented by the public sector at the time of this study: an older paper-based one and a newer computerized one initiated by the national government under the NHM in recent years. The limited data shared by a few private facilities was flowing into the paper-based reporting system but not in the computerized HMIS at the time of this study.

Besides the lack of a supportive legal framework, other barriers to sharing MNCH data included gaps in communication and follow up by the public sector, lack of standardized systems for data maintenance and collection in both sectors, the public sector’s limited capacity for data management and utilization, private providers’ fears of information disclosure, and apprehensions regarding additional burden of reporting. The enabling factor was that most facilities were willing to share MNCH data if the health department asked them to, provided the process and formats were simple and did not overstretch their existing responsibilities, and they could be reassured of information confidentiality and protection from harassment by the public sector for reporting any adverse events.

Our findings strongly suggest that even in the absence of a legal framework, the public sector can set up an effective data sharing strategy for MNCH by developing a standardized system with simple formats and data collection procedures, by thoroughly orienting private facilities’ staff as well as public sector data management staff in all the procedures, and by effectively communicating and consistently following up on data submission every month with all MNCH related private facilities. Lessons from the more successful data sharing for ultrasounds, abortions and polio eradication further emphasize the criticality of good communication and coordination together with standardized systems and proper follow up by the public sector.

In many low- and middle-income countries the private sector plays a considerable role in healthcare services and the last two-three decades have witnessed growing research on the private provision of health services including engagement of the private sector in public health activities such as immunization and family planning (Forsberg *et al.* 2011), as well as stewardship of the private sector (Forsberg and Montagu 2014). While research interest in the private sector has grown, there may still be limited attention and recognition from governments, (Travis and Cassels 2006; Forsberg *et al.* 2011); that too of a cautious, ‘command and control’ or authoritarian type (Sood *et al.* 2011). However, our study is in line with other evidence from different types of public-private engagements which suggests that effective engagements with the private sector have relied on good communication and coordination. In Tanzania, for example, a strategy for engaging the private sector in integrated delivery of insecticide treated nets through a voucher scheme proved to be successful because of; a) consultative programme development involving all stakeholders, and b) quarterly coordination meetings of all stakeholder representatives (de Savigny *et al.* 2012). The process needs champions in the initial stages, for example, a well-known senior cardiologist from the private sector was instrumental in encouraging other private providers to participate in a scheme for low cost cardiac care to the poor in the Indian state of Karnataka (Venkat and Bjorkman 2008). However, relationships and relationship building also need to be institutionalized, in order to foster sustainable engagements. A study in Zambia reported that frequent transfers of key government personnel and a project-based, donor-driven approach in developing intervention strategies often impeded efforts towards sustainable public-private engagements (Sood *et al.* 2011). The public sector may not be sufficiently motivated to invest in long term trust building with the private sector if they perceive a public private partnership as a donor driven temporary measure.

Comprehensive mapping of the private sector (location, qualifications, training levels, facility capacity and coverage) has also been found to be important before developing an engagement strategy tailored to a specific context (Brugha and Pritz-Aliassime 2003).

Incentives could play a role in increasing private sector engagement in data sharing but need to be managed skilfully. Incentives in other types of public private partnerships have included the provision of logistics and supplies, such as free or subsidized drugs, equipment and vaccines; information education and communication materials; and maintenance of equipment related to national health programmes (Kapilashrami *et al.* 2008). However, incentives alone may not work, neither do they influence everyone positively. The Revised National TB Control Programme for tuberculosis control provides a variety of incentives to private providers but has not succeeded in getting them to refer all of their tuberculosis patients to DOTS centres (Pradhan *et al.* 2011). Moreover with respect to data sharing, incentives may not be enough to get all private facilities to report regularly and consistently, and in the absence of a proper legal framework, it would be difficult for the public sector to introduce disincentives such as penalties. Any strategy needs to keep these limitations in mind.

Lessons from successful partnerships further suggest that engaging private providers in disease specific services may be easier than getting them involved in a wider range of services. The involvement of private practitioners in tuberculosis control in many countries is an example of this focused engagement (Floyd *et al.* 2006; Travis and Cassels 2006).

The novelty of our study is that it highlights the efforts that are required to be put in by the public sector if they are to engage with the private sector. The onus is as much on the public sector to create stronger and more streamlined systems for data sharing, as it is on the private sector to be more cooperative.

Our second novel finding is that a large number of institutional deliveries may be happening in unregistered facilities which are managed by informal providers. A number of Indian studies provide evidence about the presence of solo informal providers in India who are first contact providers for common child and adult illnesses (Gautham *et al.* 2014; May *et al.* 2014). A study of 108 tuberculosis patients at hospital based DOTS centres in Delhi found that 67 patients (two-thirds) had sought first treatment from informal providers and less than one-third had approached qualified providers first (Kapoor *et al.* 2012). However, we are not aware of studies that profile an informal sector in institutional delivery care (different from home births assisted by informal providers), and our study is probably among the first to suggest the existence of such a sector in institutional deliveries.

Further research exploring the role and presence of such facilities is called for and it may be worthwhile for the government to consider ways of engaging with these facilities for data sharing and service delivery. It is true that current legal frameworks appear to impede the process of engaging with unregistered facilities, but states in India are finding ways to circumvent legal barriers in public interest. For example, the Government of India (GOI) issued a government order to all states approving the involvement of AYUSH practitioners in Reproductive, Maternal, Newborn and Child Health services, especially skilled attendance at birth (GOI, 2014), so facilities with only AYUSH providers could also be licensed in future. The state government of UP recently decided to allow AYUSH practitioners to use allopathic drugs in a limited way (Times of India 2015). The state of Andhra Pradesh in south India, developed a programme of Community Paramedic training in 2008 for informal village practitioners in the state and registered them in a State Paramedical Council as a mark of formal recognition. In return the providers would refrain from calling themselves doctors (Gautham *et al.* 2014). So non-biomedical cadres are increasingly being recognized for their role in increasing access to essential health services, and states in India have chosen to adopt their own alternatives to best meet their public health goals and needs.

However, the nationwide process of developing legal and regulatory frameworks that will mandate data sharing by the private sector has only just begun in India with the passing of the Clinical Establishments Act in 2010. The Act has yet to be adopted by most Indian states including UP. Its implementation will require a substantial amount of effort and hand holding for both the public and private sectors in the coming years, and our study findings can provide useful guidance on the way forward to create a harmonious data sharing partnership.

Our study was limited to only two districts of UP (which has 75 districts), and this was a major study limitation. However we selected these two districts carefully from the 25 districts where the UP-TSU was working, using existing mapping data on the number of small and big facilities. At the district and block levels, we validated our selection of facilities by triangulating data obtained through records and through discussions with staff at block government facilities as well as at local pharmacies and small clinics. Through our systematic district selection we have tried to factor in district level variations and through our selection of facilities we have tried to include sufficient facilities with variation in bed strengths, reporting relationships and locations (rural/urban).This way we hope to increase the reliability and representativeness of our findings.

## Conclusion

Our study findings emphasize that there is definite evidence of the private sector’s willingness to share public health data that can be effectively harnessed through better communication, trust and relationship building with the public sector, and by establishing an easy, systematic and well-coordinated process of data collection and synthesis, supported by creative incentivising. More research and different solutions are required to address the needs of unregistered facilities.

## Funding

This work was supported by IDEAS - Informed Decisions for Actions to improve maternal and newborn health (http://ideas.lshtm.ac.uk), which is funded through a grant from the Bill and Melinda Gates Foundation to the London School of Hygiene & Tropical Medicine (Gates Global Health Grant Number: OPP1017031).

*Conflict of interest statement.* None declared

## Notes

The Indian Parliament passed the Clinical Establishments Act in 2010 and this new legislation will make it mandatory for all clinical establishments—public and private—to conform to prescribed quality standards, share data on nationally required parameters, display pricing, and be subjected to routine prescription audits. The Clinical Establishments Act has yet to be adopted and implemented by all the states and the centre is urging states to move ahead. In UP, existing regulation is limited to a mandatory registration of health facilities in the district Chief Medical Officer’s office. The Indian Medical Association in UP has obtained a High Court stay on periodic renewal of this registration, and so it is a one-time registration in most districts. Other active legislation (in India and in UP) includes the Post Natal Diagnostic Test Act to prevent sex determination tests that lead to sex selective abortions, and the Consumer Protection Act to protect patients against any wilful medical negligence or malpractice.
